# Gas Adsorption Investigation on SiGe Monolayer: A First-Principle Calculation

**DOI:** 10.3390/s20102879

**Published:** 2020-05-19

**Authors:** Xiang Sun, Yuzheng Guo, Yan Zhao, Sheng Liu, Hui Li

**Affiliations:** 1 The Institute of Technological Sciences, Wuhan University, Wuhan 430074, China; johnsunx@whu.edu.cn (X.S.); yguo@whu.edu.cn (Y.G.); yan2000@whu.edu.cn (Y.Z.); li_hui@whu.edu.cn (H.L.); 2 DOE Lab for Hydropower Transients, School of Power and Mechanical Engineering, Wuhan University, Wuhan 430072, China; 3 School of Electrical Engineering, Wuhan University, Wuhan 430072, China; 4 Research Institute of Wuhan University in Shenzhen, Shenzhen 518057, China

**Keywords:** first-principles, 2D monolayer, gas adsorption

## Abstract

The gas adsorption behaviors of CO, CO_2_, SO_2_, NO_2_, NO, NH_3_, H_2_, H_2_O, and O_2_ on SiGe monolayer are studied using the first-principles calculation method. Three special adsorption sites and different gas molecule orientations are considered. Based on adsorption energy, band gap, charge transfer, and the electron localization function, the appropriate physical adsorptions of SO_2_, NO, NH_3_, and O_2_ are confirmed. These gases possess excellent adsorption properties that demonstrate the obvious sensitiveness of SiGe monolayer to these gases. Moreover, SiGe may be used as a sensing material for some of them. NO_2_ adsorption in different adsorption sites can be identified as chemical adsorption. Besides, the external electric field can effectively modify the adsorption strength. The range of 0 ~ − 2 V/nm can create a desorption effect when NH_3_ adsorbs at the Ge site. The NH_3_ adsorption models on Ge site are chosen to investigate the properties of the I-V curve. Our theoretical results indicate that SiGe monolayer is a promising candidate for gas sensing applications.

## 1. Introduction

With the increasing concern about environment protection, the demand for gas sensing devices has increased. However, there are several shortcomings of the existing gas sensing devices, such as low sensitivity [1], poor reusability [2], the drift of sensitivity [3], and sensing efficiency [4]. Because of these, it is a matter of utmost urgency to find suitable new kinds of sensing materials. The unique electronic properties, large specific surface area, and high mechanical properties make two-dimensional (2D) materials become the research focus [5]. Many 2D materials have been investigated as chemiresistors, FETs sensors, and optical sensors such as graphene oxide, MoS_2_, and phosphorene [6]. For the sake of the large surface-to-volume ratio and high charge carrier concentration, 2D material can be a good candidate for gas sensors [7].

Different from graphene, which consists of only one type of element, a single layer material composed of two types of elements is also of great interest, including planar silicon carbide [8] (SiC), which opens a band gap to become semiconducting. In this paper, low-buckled silicon germanium [9,10] (SiGe) with a band gap is our research focus. SiGe monolayer has been discussed as a ferromagnetic material, as presented by Zhou et al. [9]. Juarez-Mosqueda et al. investigated a halogenated SiGe monolayer as a topological phase transition [11]. The lattice constant of this material is 3.95 Å, which is nearly the average of the lattice constant of 3.86 Å and 4.0 Å for silicene [12,13] and germanene [13], respectively. The bond length of Si – Ge is 2.32 Å. The Dirac cone presents at the K point where the π and π* bands consist of the p_z_ orbitals of Ge and Si atoms. A band gap of nearly 0.02 eV exists at the Dirac cone. Moreover, the higher energetic and kinetic stability make SiGe monolayer easier to obtain compared with germanene through the previous computation.

In this work, the results of theoretical studies based on density functional theory on SiGe monolayer gas adsorption are presented. Several common pollutant gases including CO, CO_2_, SO_2_, NO_2_, NO, NH_3_, H_2_, H_2_O, and O_2_ are considered in this paper. The adsorption energy (*E*_ad_), most preferential adsorption site, and gas molecule direction are discussed firstly. At the same time, the interaction between the gas molecule and SiGe monolayer is also an important research point. When the most suitable adsorption site is selected, adsorption distance, partial density of states (PDOS), and charge transfer between the two parts are employed to discover the mechanism. As an assistive method, the charge density difference and band gap modification analysis are also calculated. In order to consider the electric field influence, the adsorption model is calculated under an external electric field. Our studies find that SiGe monolayer demonstrates impressive adsorption performance for some particular gases compared to other 2D materials such as too large adsorption energies of SO_2_ (−5.26 eV) and NO_2_ (−4.61 eV) adsorbed on AlN monolayer [14].

## 2. Calculation Methods

The electronic and structure calculations were performed using the numerical basis set as implemented in the Dmol^3^ code [15]. The generalized gradient approximation (GGA) with the Perdew–Burke–Ernzerhof (PBE) parametrization was used in the calculation [16]. The ultra-soft pseudopotential was adopted during the calculation, while the Grimme custom method was also used for vdW (van deer Waals) correction [17] (the semi-empirical dispersion interaction correction with the Grimme method was adopted to treat the van der Waals interaction of the adsorption system). To carry out the Brillouin-zone integration, a high Monkhorst–Pack k-point grid parameter was employed as 21 × 21 × 1, while for the density of states calculation, the parameter was set as high as 25 × 25 × 1 in order to acquire a higher calculation accuracy. The effective core and double numeric with polarization (DNP) were selected. The forces on all atoms were less than 0.05 eV/Å. The global orbital cutoff radius was set to be 5.0 Å. All the computational parameters mentioned above were carefully tested to achieve convergence of the total energy [18]. Besides, for some polar gas molecules like NO_2_, the spin unrestricted set was used in Dmol^3^ code.

A 4 × 4 supercell (32 atoms) of SiGe monolayer was used with the lattice constant of a = b = 15.67 Å, which was in good agreement with previous reports [9]. To avoid interactions between adjacent layers and the adsorbed gas molecule, a vacuum space of 15 Å was introduced along the direction perpendicular to the 2D plane. To evaluate the stability of the SiGe monolayer adsorption system in different gas molecules, the adsorption energy (*E*_ad_) is defined as [19]:

$$E_{\mathrm{ad}}=E_{\mathrm{SiGe}+\mathrm{gas}}-E_{\mathrm{SiGe}}-E_{\mathrm{gas}}$$

where $E_{\mathrm{SiGe}+\mathrm{gas}}$ is the total energy of the adsorbed gas molecules on the SiGe monolayer system; moreover, $E_{\mathrm{SiGe}}$ and $E_{\mathrm{gas}}$ are the total energies of pure substrate monolayer and the isolated gas molecule, respectively. By definition, negative *E*_ad_ corresponds to favorable or exothermic adsorption of gases on the SiGe monolayer system.

## 3. Results and Discussion

**Figure 1:**
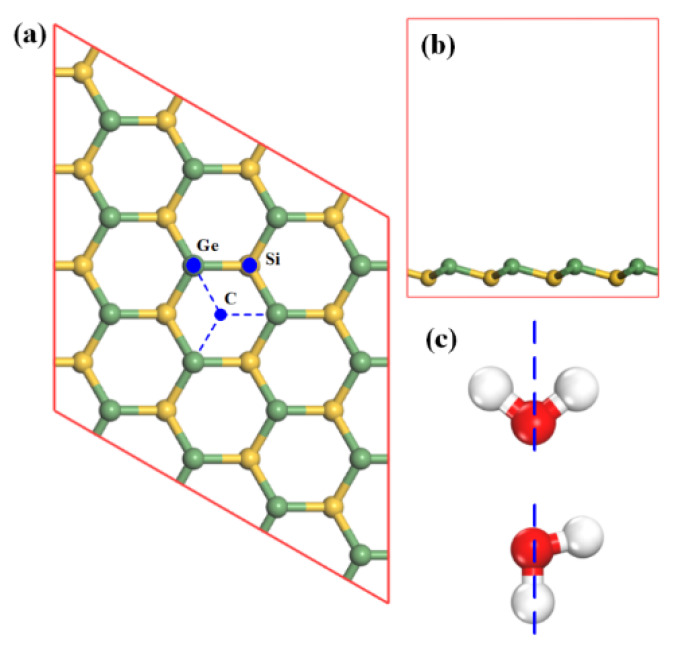
(**a**) Schematic view of the geometric structure for 4 × 4 SiGe monolayer with three selected adsorption sites (Ge atom site, Si atom site, and center site of hexagon); (**b**) the side view of SiGe monolayer; and (**c**) gas molecule orientations (taking the H_2_O molecule as an example).

In order to find the most stable adsorption configurations, the gas molecule was placed at three selected positions above SiGe monolayer with different orientations [20]. As illustrated in Figure 1a, the three selected sites, namely Ge site, Si site, and center (C) site, were considered. The Ge site was directly above the Ge atom, the Si site directly above the Si atom, and the C site at the center of a hexagon composed by Ge and Si atoms. Intuitively, when the molecule was adsorbed on the substrate, different gas molecule orientations (presented in Figure 1c) would produce different adsorption effects. The situation also appeared in other gases’ adsorption [21]. Therefore, in this work, the effect was also considered. In Figure 1c, the blue dashed line represents the *z*-axis where the lower atoms would be placed close to the substrate. For the sake of brevity and calculation source limitation, guaranteeing the accuracy of calculation at the same time, the paper just considers single gas molecule adsorption.

To explore the different adsorption effects between SiGe substrate and calculated gas molecules including CO, CO_2_, SO_2_, NO_2_, NO, NH_3_, H_2_, H_2_O, and O_2_, the detailed information about the most stable adsorption sites is provided in Table 1, containing equilibrium substrate-gas distance (*D*), adsorption energy, and charge transfer (*Q*). *D* is defined as the center-to-center distance of the nearest atoms between SiGe and the gas molecule; *Q* is defined as the total Mulliken charge transfer between the model SiGe monolayer and the gas molecule, and a negative number means charge transfer from SiGe to the gas molecule.

### 3.1. CO, CO_2_, H_2_, and H_2_O Adsorption

For CO and CO_2_ on SiGe, the adsorption effects were obviously different for different adsorption sites. The largest region for CO was around −0.24 eV, while the largest *E*_ad_ for CO_2_ was −0.2 eV, which meant the SiGe monolayer was slightly more sensitive to CO. Then, for CO adsorption, the C orientation (the C atom points directly to the SiGe substrate) was more suitable than the O orientation adsorption models (O points directly to the adsorption site). The most stable adsorption site of CO was the center site. The shortest distance (*D*) was 3.41 Å, which existed between the C and Ge atoms. The considerably large *D* corroborated the smallest value of *E*_ad_. Moreover, after the adsorption, electrons transferred from the substrate to CO, which may induce a doping behavior. The most stable adsorption site for CO_2_ was different. The smallest *E*_ad_ of −0.2 eV emerged in the Si site when the C atom of the gas molecule pointed to the substrate. The adsorption induced a larger *E*_ad_ compared to that of CO adsorption. The distance between C and Si was 3.89 Å, and the amount of electrons that transferred to the gas molecule was smaller than the CO adsorption, which was consistent with the adsorption energies.

The H_2_ adsorption at the center site showed an *E*_ad_ as −0.15 eV, which was really small to make us believe that it was a physical adsorption. The electrons transferred from the substrate to the H_2_ molecule were few, and the distance was relatively large, presenting as 2.89 Å between the center to the H atom, the distance between the H and Ge/Si atoms being even larger, at least 3.66 Å (H-Ge). For H_2_O adsorption, *E*_ad_ = −0.35 eV was a relatively suitable physical adsorption value for gas detecting. The O and Ge distance was 3.00 Å, which makes building the covalence bond difficult. The electrons moved from the gas molecule to the substrate, and the large amount of electrons (0.03 |*e*|) just corresponded to the relatively large adsorption energy.

### 3.2. NO, SO_2_, and NH_3_ Adsorption

For NO adsorption, the center and Ge sites presented the most attractive adsorption performances with the N atom of NO molecule all closing to the Ge atom. The center site exhibited a smaller value of *E*_ad_ of −0.48 eV, and the shortest distance existed between the N and Ge atoms, which reached 2.80 Å. Meanwhile, the Ge site presented a slightly smaller absolute value of *E*_ad_ of 0.40 eV. These two values seemed to be in attractive physical adsorption ranges. To ensure the preliminary guess, the adsorption distance was also explored. In order to consider the possible built bond, the distance between N and the atom around the hexagon was investigated. The shortest distance of N and Ge is mentioned above. Regarding the covalent radius, the N covalent radius was 0.71 Å, and that of Ge was 1.20 Å. The existing N-Ge distance (2.80 Å) was larger than the covalent radius sum (1.91 Å) of separate N and Ge atoms, which might indicate that the adsorption could be a kind of physical adsorption. Charge transfer demonstrated that the NO molecule obtained charge as 0.03 |*e*|. For Ge site adsorption of NO, the adsorption energy was −0.40 eV, and the *D* was 2.28 Å (distance of N-Ge). The *D* of the Ge site was smaller than that of the center site; however, the center site adsorption exhibited a smaller *E*_ad_ and a smaller amount of |*Q*|. This may be attributed to the center adsorption site that can be influenced by too many atoms surrounding especially the three nearest Ge atoms. Every Ge atom of the hexagonal ring contributing to the adsorption made the *D* relatively average to Ge atoms and may also reduce the charge transfer from SiGe to NO. Considering the calculated parameters, the physical adsorption could be verified for NO adsorbing on the center and Ge sites.

Similar to the NO adsorption, there were two sensitive sites for SO_2_ adsorption that drew our attention. The Ge and Si sites with the S atom pointing directly to them initially possessed two attractive values of adsorption energy as −0.57 and −0.78 eV, respectively. The S-Ge distance was 2.76 Å of Ge site adsorption. For Si site adsorption with S pointed to, the shortest distance became O-Ge as 2.11 Å, while the distance of S-Si was 3.61 Å. This adsorption was accomplished with obvious Si site deformation. However, the *E*_ad_ of SO_2_ adsorbed on the Ge site with the O atom pointed to was just −0.11 eV with a distance of 3.19 Å for O-Ge. This interesting scene could be explained through the adsorption structure. When the calculation is ready for S pointing directly to the Si site, the Si atom of SiGe monolayer became deformed. Si seemed to be repelled by the S atom, inducing bond elongation between Si and Ge; at the same time, the O atom gradually became close to the Ge atom, and SO_2_ finally became almost parallel to the SiGe plane, which was different from Figure 1c. The two sites adsorptions showed a large number of electrons transferring to SO_2_, and physical/chemical adsorptions might exist, respectively. That will be investigated later.

For NH_3_ adsorbed at SiGe monolayer, the three adsorption models were all calculated, and two larger adsorption energy models were selected as the Ge and Si sites. The same adsorption energies of −0.62 eV excited our interest. More importantly, it was an attractive value for physical adsorption as a gas sensing application. Hence, the calculation results were tested, and it was found that the NH_3_ placed at the Si site moved to the Ge site finally. Therefore, the Ge site must be the most attractive sensing site for NH_3_. The distance between N and Ge was 2.32 Å, which was larger than the covalent radius sum (1.91 Å) of separate N and Ge atoms. As a consequence of all the above, NH_3_ adsorption on the Ge site might be physical adsorption with a moderate adsorption energy.

### 3.3. NO_2_ and O_2_ Adsorption

In the case of NO_2_ adsorption, the most energetic favorable site where the NO_2_ molecule preferred to reside was the Ge and Si sites. After full relaxation, the N and O atoms all preferred to point closely to SiGe monolayer. The adsorption energies for N close to the Ge site, O close to the Ge site, O close to the Si site, and N close to the Si site were −1.00 eV, −1.14 eV, −1.140 eV, and −1.24 eV, respectively. The super large adsorption energies made us pay attention to all these adsorption sites. When the O atom of NO_2_ pointed to the Ge and Si sites, the same adsorption energy made us wonder if the situation was similar to the NH_3_ adsorption mentioned above. Through our calculation, the O atom firstly pointing directly to the Si site would move to the nearest Ge site and finally kept the same distance (2.03 Å) of Ge-O compared to the situation of O pointing directly to the Ge site. This indicated that the Ge site was the most sensitive site when the O of NO_2_ pointed directly to SiGe monolayer. As for the N of NO_2_ pointing to the Ge site and Si site, the adsorption energies were still very large as −1.00 eV and −1.24 eV. The distances of the two situations were 2.16 Å and 1.96 Å, respectively. The shortest distance corresponded to the largest adsorption energy, which simply confirmed the correctness of our work. As for the adsorption model, the covalent radius of N-Si was 1.82 Å, which is very close to the 1.96 Å of the largest *E*_ad_ adsorption (Si site adsorption with N atom pointing to it). The largest adsorption energies and small distances might imply chemical adsorption activities; meanwhile, the physical or chemical adsorption species of the other NO_2_ adsorptions were hard to determine considering the large *E*_ad_ and relatively small distances (the covalent radii of O-Ge and N-Ge were 1.86 Å and 1.91 Å, respectively). The charges moved from SiGe to NO_2_, reaching about 0.3 ~ 0.4 |*e*|. Analyzing the band gap of NO_2_ adsorption, the Fermi level moved down to the valence band, which showed a p-type doping. As for O_2_ adsorption, the adsorption energy was −0.93 eV; meanwhile, the shortest distance between O_2_ and SiGe monolayer was the O-Ge distance, which was 2.93 Å. As mentioned above, the covalent radius of O-Ge was 1.86 Å, which was nearly 1 Å smaller than that of O_2_ adsorption. It was hard to distinguish the adsorption type because of the large adsorption energy and relatively large atom distance. The uncertain gas adsorption models (physical/chemical adsorption) of NO_2_ and O_2_ will be studied later.

**Figure 2:**
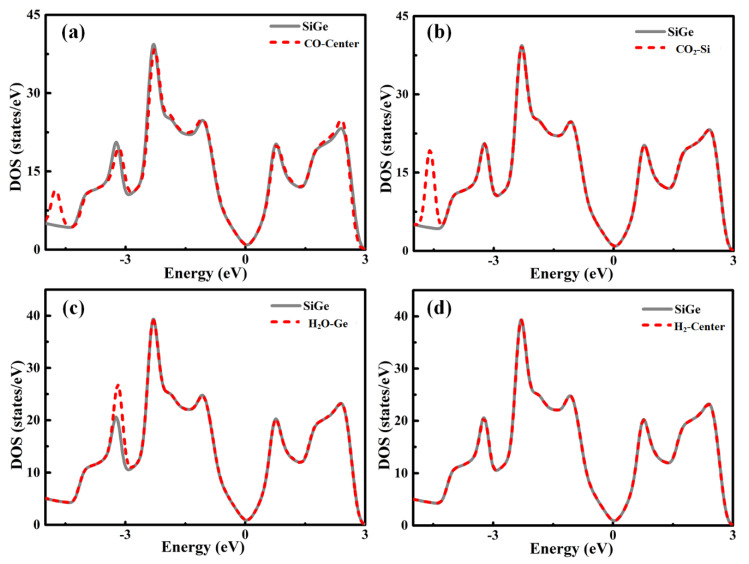
Total density of state for the (**a**) CO-center, (**b**) CO_2_-Si, (**c**) H_2_O-Ge, and (**d**) H_2_-center adsorption models. DOS, density of state.

In order to explore the fundamental interaction of Gas-SiGe adsorption, the total electronic density of states is presented in Figure 2 [22]. The spin polarization effect was considered for some specific gases, and the results indicated that there were no significant spin polarizations in the total density of state (DOS) plots. Therefore, the DOS plots all adopted no spin polarization effect pattern.

The DOS plots of CO, CO_2_, H_2_O, and H_2_ adsorption are presented in Figure 2a–d, respectively. From the pictures above, these gases’ adsorptions induced some changes in the trend of the DOS lines. However, during the critical area around the Fermi level, the lines of separate SiGe and gas-SiGe systems fit each other perfectly. This phenomenon suggested that the interaction between the gas molecule and SiGe was negligible. This phenomenon was the same as the result of physical adsorption. These results revealed the same conclusion as the adsorption energy and charge transfer.

**Figure 3:**
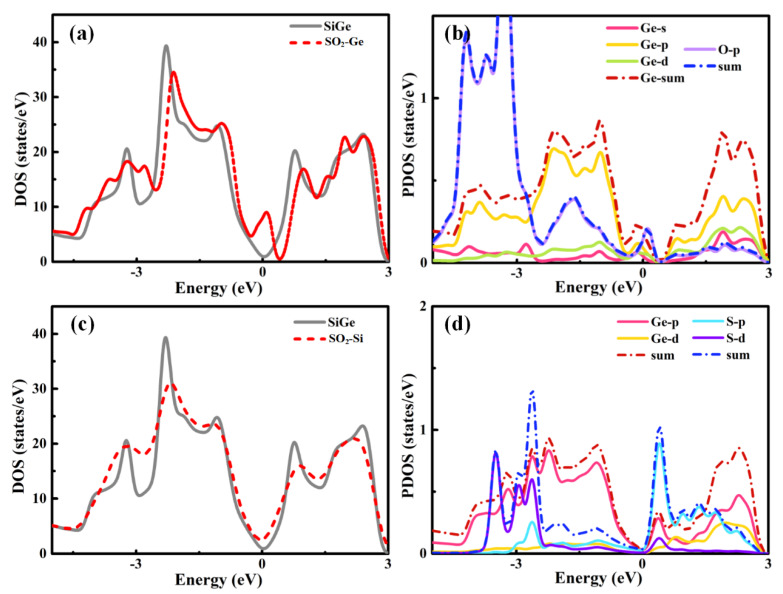
Total and partial density of states for (**a**), (**b**) SO_2_-Ge and (**c**), (**d**) SO_2_-Si adsorptions.

Analyzing the DOS plots of SO_2_ in Figure 3, the differences between SO_2_ and those above were clear. The obvious difference around the Fermi level between the separate SiGe monolayer and SO_2_-SiGe system presented a relatively strong interaction between the gas molecule and substrate material. Ge site adsorption induced a small peak at the right side of the Fermi level and the little shifts of DOS toward VBM corresponding to the p-type doping that corresponded to the charge transfer for SiGe to the SO_2_ molecule as 0.29 |*e*|. The p orbital of Ge and p orbital of S shared a hybridization and contributed to the small peak near the Fermi level. In the meantime, the Si site adsorption induced a large peak around the Fermi level, and the DOS shift seemed more serious than Ge site adsorption with larger charge transfer to the SO_2_ molecule as 0.477 |*e*|. The partial density of states (PDOS) plot implied that the p and d orbitals of Ge and the p orbital of O led to the strong interactions.

**Figure 4:**
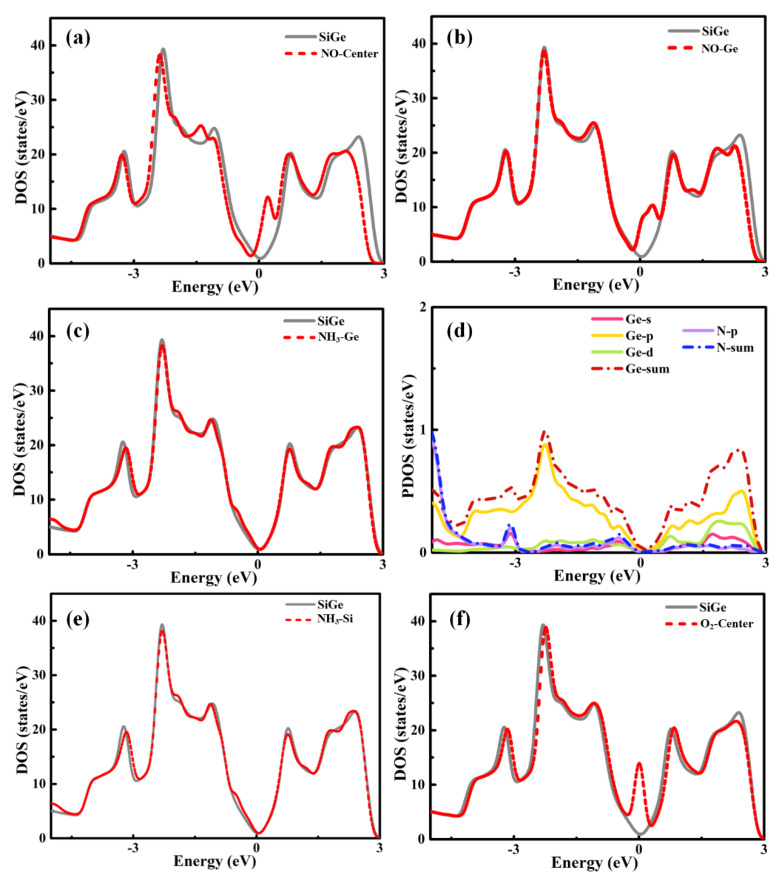
Total density of states for (**a**) NO-center, (**b**) NO-Ge, (**c**) NH_3_-Ge, (**d**) partial density of states (PDOS) for NH_3_-Ge adsorptions, and (**e**) NH_3_-Si, (**f**) O_2_-center.

For NO, NH_3_, and O_2_ adsorption, the DOS and PDOS figures are presented in Figure 4. The NO adsorptions on SiGe monolayer at the center and Ge sites caused quite an increase of DOS around the Fermi level. This implied the relatively strong interaction between the gas molecule and SiGe monolayer, which was similar to SO_2_ adsorption. The slight movement of the Fermi level, especially N-center adsorption, was in good agreement with the charge transfer to the gas molecule. When NH_3_ was adsorbing in SiGe monolayer, the same final adsorption site mentioned above was confirmed again through the same DOS plots in Figure 4c,e. The PDOS plot announced that the s, p, and d orbitals of Ge plus the p orbital of N mainly contributed to the attractive adsorption, while the s and p orbitals of Ge together with the p orbital of N contributed to the slight increase of the valence band gap area near the Fermi level. The DOS in Figure 4f of the O-SiGe model was consistent with the strong adsorption behavior. A strong peak appearing around the Fermi level suggested the intense interaction in the adsorption. According to the small distance and large adsorption energy, it must be a chemical adsorption.

**Figure 5:**
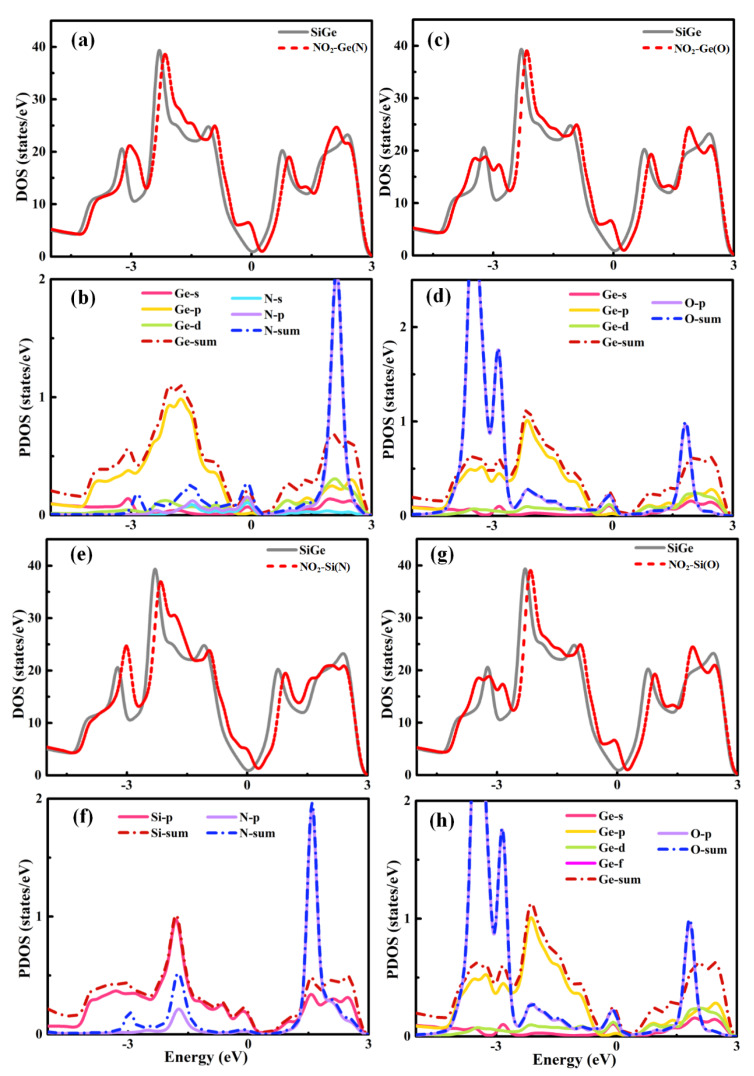
Total/partial density of states for (**a**),(**b**) NO_2_-Ge(N), (**c**), (**d**) NO_2_-Ge(O), (**e**), (**f**) NO_2_-Si(N), and (**g**), (**h**) NO_2_-Si(O) adsorptions (Figure a,c,e and g are total density of states; Figure b,d,f and h are partial density of states).

Taking into account the four situations of NO_2_ adsorption, the plots of b, d, f and g are the PDOS patterns of Figure 5. Figure 5a,b,e,g are the DOS patterns. First, the same DOSs of O pointed directly to Ge and Si, respectively, implying that the final adsorption structures of the two situations became the same one: the O atom pointing to the Ge site adsorption model (shown in Figure 5c,d,g,h). Thus, the DOS plots confirmed the result mentioned above. The peaks around the Fermi level during these situations showed the interaction. Moreover, the relatively small peak, shown in Figure 5e, of the Si site adsorption (N orientation) during the four situations related to the smallest adsorption energy. The similar Fermi level movements implied p-type doping where the charges transferred to the gas molecules. By analyzing the other figures in detail, Figure 5a,b show that the s and d orbitals of Ge together with the s and p orbitals of N contributed to the large increase in the Fermi level, which was consistent with the largest adsorption energy. At the same time, the pretty large increase in Figure 5c,g for O-Ge adsorption patterns was mainly induced by the s and d orbitals of Ge coupled with the p orbital of O. The interaction between the gas molecule and substrate layer was slightly smaller than that in Figure 5a.

### 3.4. Charge Density Difference

**Figure 6:**
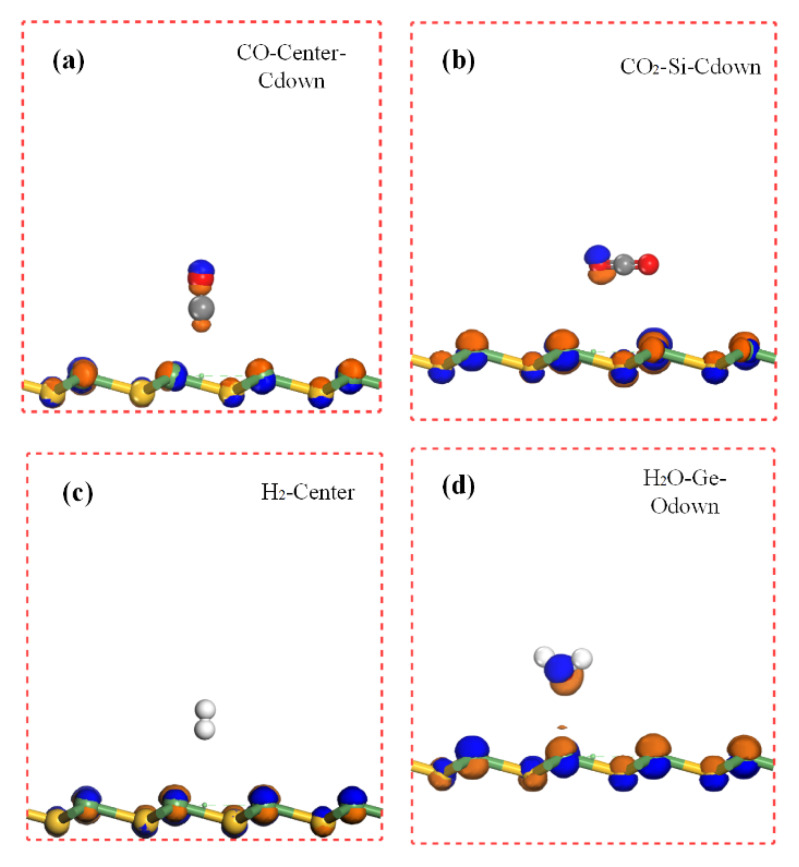
The charge density difference of (**a**) CO, (**b**) CO_2_, (**c**) H_2_, and (**d**) H_2_O adsorbed on SiGe monolayer (the contour lines in plots are drawn at 0.02 e/Å^3^ intervals). The blue and brown areas represent accumulation and depletion of charge density.

**Figure 7:**
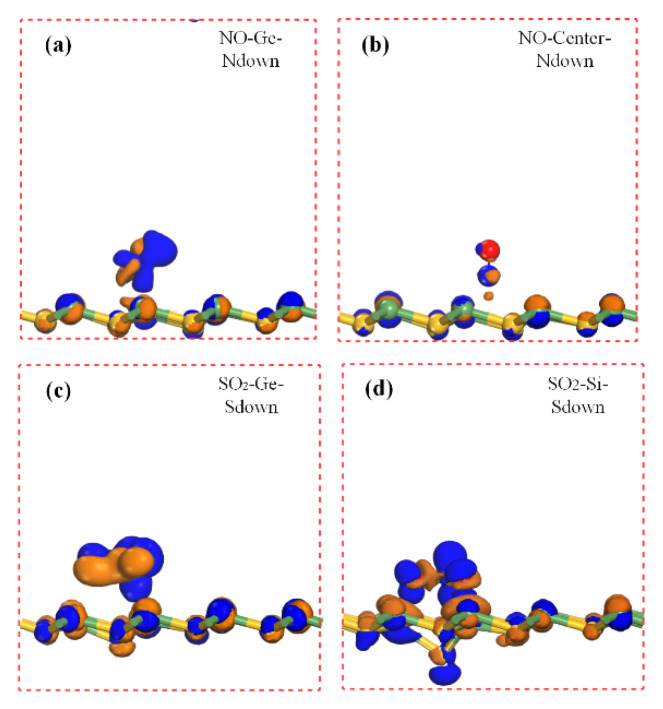
The charge density difference of NO (Figure (**a**) shows NO on Ge site and (**b**) shows NO on Center site) and S_2_O (Figure (**c**) shows SO_2_ on Ge site and (**d**) shows SO_2_ on Si site) adsorbed on SiGe monolayer.

**Figure 8:**
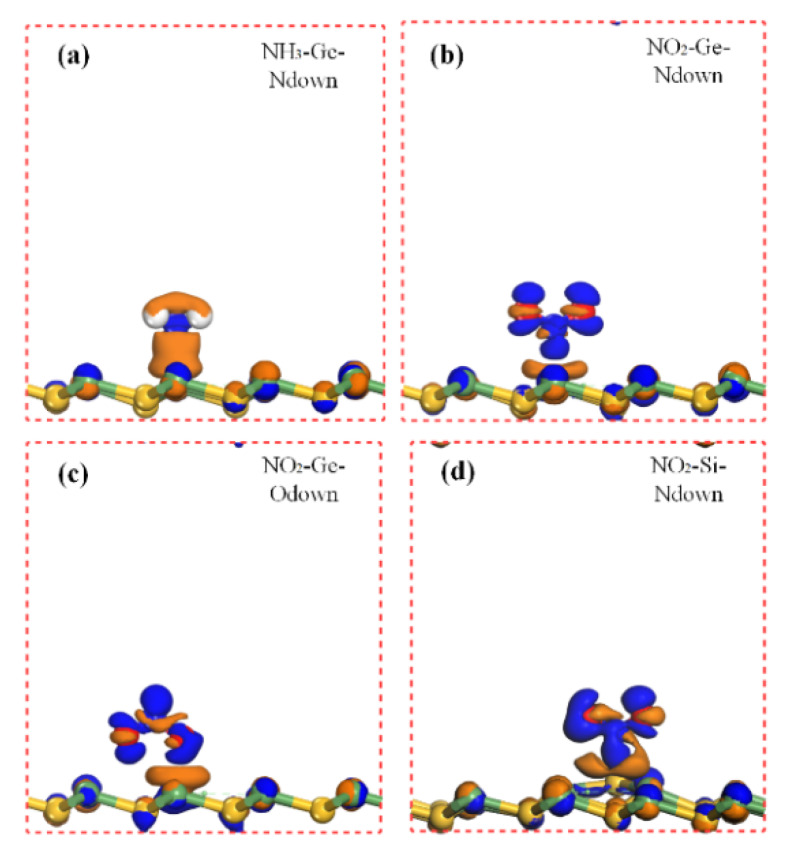
The charge density difference of NH_3_ (Figure (**a**) shows NH_3_ on Ge site) and NO_2_ (Figure (**b**) shows NO_2_ on Ge site with N pointed, (**c**) shows NO_2_ on Ge site with O pointed, and (**d**) shows NO_2_ on Si site) adsorbed on SiGe monolayer.

Charge transfer is an important parameter to determine the interaction between the gas molecule and the substrate material. The charge density difference (CDD) [23] can intuitively help us figure it out. Figure 6, Figure 7 and Figure 8 elucidate the CDD images of these Gas-SiGe systems, calculated by the formula [24] *△ρ* = *ρ*_SiGe+molecule_ – (*ρ*_SiGe_ + *ρ*_molecule_), where *ρ*_SiGe+molecule_, *ρ*_SiGe_, and *ρ*_molecule_ are the charge density of the gas-adsorbed SiGe monolayer, the pristine SiGe monolayer, and the gas molecule, respectively. *ρ*_SiGe_ and *ρ*_molecule_ must be calculated in the same adsorbed configuration.

The charge redistributions caused by gas adsorptions are clearly described in Figure 6, Figure 7 and Figure 8. After CO adsorption, the charge transfer apparently moved from SiGe to the CO molecule analyzed in Table 1. At the same time, the CDD plot in Figure 6a also verified this, which implied a charge redistribution in the CO molecule. The charges concentrated in the O atom. The CO_2_ adsorption exhibited smaller charge transfer to the CO_2_ molecule mainly remaining in the O atoms. Moreover, the charge movement in H_2_ adsorption was comparably small, so that there was barely any charge image in the H_2_ molecule under this isosurface value. For H_2_O adsorption, there was a positive charge state in H_2_O after adsorption, while the O atom owned the sole negative charge. For NO adsorption at center and Ge sites, the negative charge scattered on the N and O atoms. Considering the SO_2_ adsorption, the charge redistribution was obviously larger than other gas adsorption mentioned above. Since the Si site adsorption (Figure 7d) caused obvious SiGe monolayer deformation. The positive and negative charge redistributions between O atoms and Ge atom demonstrated the bond existing as chemical adsorption. However, the comparatively little charge redistribution displayed a smaller adsorption strength. It was still hard to confirm the adsorption style for SO_2_-Ge (the S atom pointed to the Ge site) adsorption, and it will be ascertained in the next part. A similar situation was also found when NH_3_ adsorbed on SiGe at the Ge site with N pointing directly to the Ge atom (shown in Figure 8a). Many electrons are depleted in NH_3_ and the area between the gas molecule and SiGe monolayer. H atoms carry a positive charge, and the negative charges concentrated on the N atom at the same time. To date, NO_2_ adsorption style has been identified as chemical adsorption. The CDD plots and Mulliken charge analysis shared similar results that the NO_2_ molecule totally carried a negative charge and a strong charge transfer occurred between the gas molecule and the nearest atoms of the substrate monolayer.

### 3.5. Electron Localization Function

**Figure 9:**
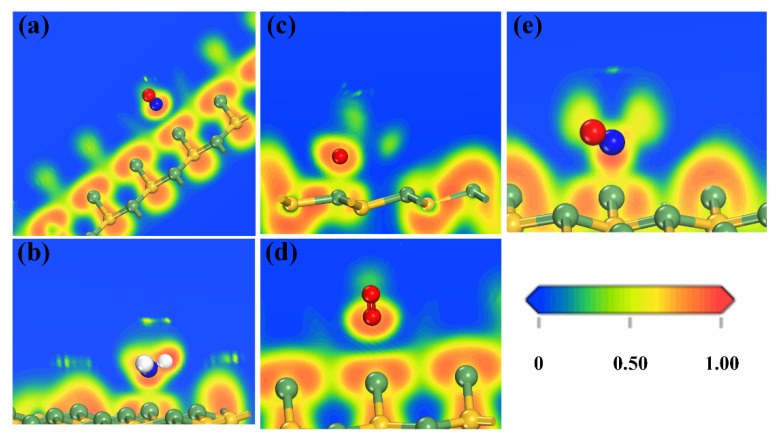
Electron localization function profiles of (**a**) NO-center, (**b**) NH_3_-Ge, (**c**) SO_2_-Si, (**d**) O_2_-center, and (**e**) NO_2_-Ge sites’ adsorptions.

In order to confirm the bond existing between some high adsorption energies and short distance adsorption models, the electron localization function (ELF) [25,26,27] was used. ELF is an effective tool for understanding the characteristics of chemical bonding and the lone electron pairs (non-bonding electron pairs) by qualitatively describing the degree of electron localization. The function has a value between zero and one, where ELF = 1 indicates the prefect localization characteristics of the covalent bonding or the lone electron pairs, ELF = 0.5 represents the electron-gas-like pair probability, and ELF = 0 corresponds to delocalization. For brevity, NO-center, NH_3_-Ge, SO_2_-Si, O_2_-center, and NO_2_-Ge (N pointed to) were considered. For NO-center adsorption shown in Figure 9a, the value between N and the nearest Ge atom was smaller than 0.5, close to zero. There was no bond between N and the nearest Ge. For NH_3_ and SO_2_ adsorption shown in Figure 9b,c, the situations were different. The values were larger than 0.5, which represented the electron localized and the interactions between gas molecules and substrate atoms were strong. The new bonds were built during the two gases’ adsorption. They were considered as chemical adsorption. As shown in Figure 9d, O_2_ adsorption at the center site showed an obvious separation between the O and Ge area in the ELF plot. The nearly zero value of ELF is shown as the blue area, and electrons were evenly distributed around the O and Ge atoms, which ensured that O_2_ adsorbed at the center site in a physical adsorption model. However, NO_2_ adsorption at the Ge site with the N atom orientation possessed the smallest adsorption energy compared with other NO_2_ adsorptions. If it were verified as chemical adsorption, the other NO_2_ adsorptions could be considered as chemical adsorption naturally. In Figure 9, the ELF value at the area between N and Ge was really close to one. The covalent characteristic bond was obvious. Therefore, there must be a new bond built between N and Ge when NO_2_ adsorbed at the Ge site with N pointing to it. Moreover, the other NO_2_ adsorptions mentioned above also would be chemical adsorption.

### 3.6. Effect of an Electric Field on SiGe-Gas Interaction

**Figure 10:**
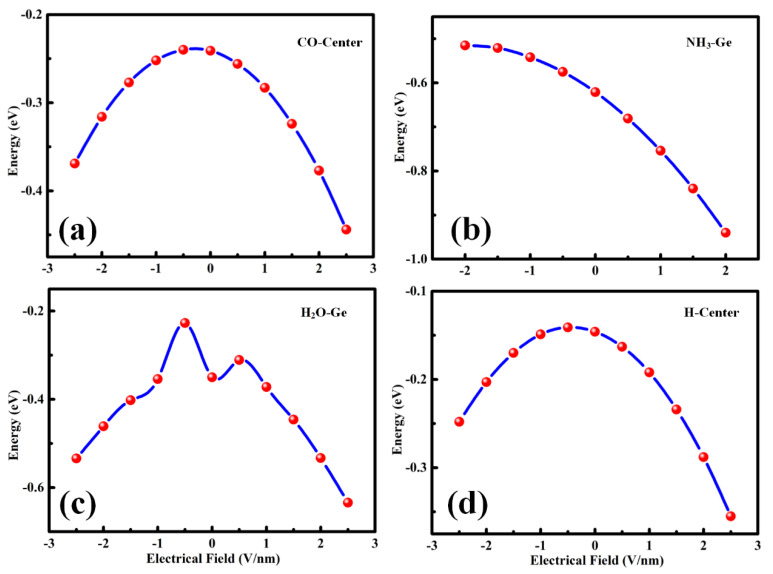
The calculated adsorption energy as a function of applied external electric field (E-field) for (**a**) CO-center, (**b**) NH_3_-Ge, (**c**) H_2_O-Ge, and (**d**) H_2_-center adsorbing at SiGe monolayer, and the external E-field is perpendicular to the plane of SiGe monolayer with its positive direction aligned upward toward the coordinate axes.

The charge transfer between the gas molecule and the monolayer material played a crucial role in interaction and adsorption strength. An external electric field is a common method to adjust the property of the material. It can effectively alter the charge transfer. Therefore, the E-field [28,29] was used to modify the adsorption effect. As shown in Figure 10, the small *E*_ad_ adsorption models of CO, H_2_O, and H_2_, plus the attractive *E*_ad_ adsorption of the NH_3_-Ge-site were calculated. The CO and H_2_ models shown in Figure 10a,d exhibited the same trend under the E-field. The E-field produced an obvious enhancement in gas adsorption. As for H_2_O adsorption, the increasing values of |E-field| in 0 ~0.5 and 0 ~ −0.5 made |*E*_ad_| decrease, and the negative direction E-field possessed a more obvious phenomenon. Then, the adsorption energy became larger when the E-field increased. Moreover, in Figure 10b, there was a different trend. The positive direction of the E-field similarly enhanced |*E*_ad_|, making the adsorption stronger, while the negative direction of E-field displayed a trend of reducing |*E*_ad_|. This was an interesting effect that might be used in the desorption process, which may improve the utilization rate of the material.

**Figure 11:**
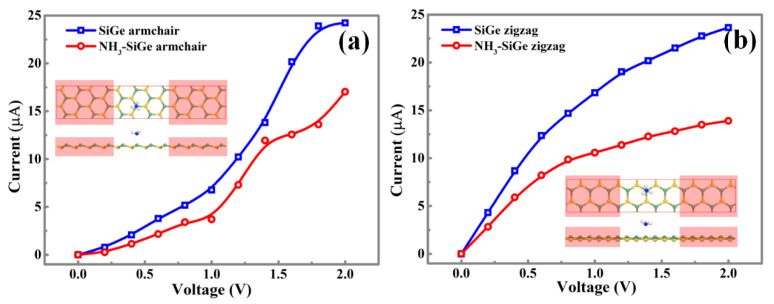
The I-V curves along the two directions of the pure SiGe monolayer and SiGe with the NH_3_-Ge adsorption. The armchair direction (**a**) and zigzag direction (**b**) are shown above.

To explore the performance of the SiGe monolayer, the NH_3_-Ge adsorption model was chosen to calculate the current-voltage [30,31,32] (I-V) relation before and after the gas adsorption through the NEGF (Non-Equilibrium Green Function) method showing in Figure 11. The armchair direction of the intrinsic SiGe monolayer showed a small irregular increase, and the curve reached the extremum at around 1.8 V. The NH_3_ adsorption made the I-V curve more irregular, but it still showed an increasing trend. For the zigzag direction of the intrinsic SiGe monolayer, the I-V curves increased along with the rising bias from 0 to 2.0 V with a decreased slope, and it would be its extremum when 2.0 V was applied. The NH_3_ adsorption in Ge site reduced the conductive ability, which might be beneficial for the application of gas sensing.

## 4. Conclusions

Using density functional theory (DFT) calculations, the adsorption behaviors of CO, CO_2_, SO_2_, NO_2_, NO, NH_3_, H_2_, H_2_O, and O_2_ adsorbed on SiGe monolayer were investigated. With the analysis of adsorption energy, charge transfer, DOS, and CDD, the gas groups of CO, CO_2_, H_2_, and H_2_O showed weaker adsorption strength, indicating a not very sensitive property for these gases. Nevertheless, for SO_2_ (Ge site), NO, and O_2_ adsorption, the physical adsorption was certain, which was verified by comprehensive adsorption energy and ELF analysis. Moreover, these gas adsorptions were in excellent agreement with the values, which ensured the adsorption strength and sensitivity. The results exhibited that the SiGe monolayer might be suitable for these gas adsorptions. SiGe monolayer exhibited pretty good sensitivity to O_2_, which made us pay more attention to the oxidation effect when the material was used. NO_2_ and NH_3_ adsorptions had super high adsorption energies. The chemical adsorption model was also made certain through ELF analysis. At the same time, SO_2_ adsorbed at the Si site could induce atomic repulsion. Then, the O atom moved close to the Ge atom and produced a strong interaction. A new bond was built between the O and Ge atoms. There were adsorption enhanced effects under the external electronic field for the gas chosen. NH_3_ (Ge site) adsorption showed a slightly different effect that the |*E*_ad_| would decrease under the range 0 ~ −2 V/nm. Finally, the I-V curves of NH_3_ adsorption were also investigated. The impurities such as low-frequency noise could be an important research point in 2D material sensing [33]. It may be useful to guide the use of SiGe monolayer, and this is worthy of further exploration. Our results implied that SiGe monolayer is promising for gas sensing applications.

## Figures and Tables

**Table 1:** The adsorption value of SiGe monolayer for CO, CO_2_, SO_2_, NO_2_, NO, NH_3_, H_2_, H_2_O, and O_2_. *E*_ad_ (eV): the adsorption energy. *Q* (*e*): the charge transfer from the gas molecule to SiGe monolayer. *D* (Å): the shortest distance of the atom in the molecule to the SiGe surface.

	*E*_ad_ (eV)	*D* (Å)	*Q* (*e*)	Bandgap (eV)
CO-center C down	−0.24	3.41 (C-Ge)	−0.01	0.01
CO_2_-Si C down	−0.20	3.89	−0.01	0.02
SO_2_-Ge S down	−0.57	2.76 (S-Ge)	−0.29	0.09
SO_2_-Si S down	−0.78	2.11 (O-Ge)	−0.48	0.12
NO_2_-Ge N down	−1.00	2.16	−0.32	0
NO_2_-Ge O down	−1.14	2.03	−0.37	0
NO_2_-Si O down	−1.14	2.03	−0.37	0
NO_2_-Si N down	−1.24	1.96	−0.40	0
NO-center N down	−0.48	2.80 (N-Ge)	−0.03	0
NO-Ge N down	−0.40	2.28	−0.06	0
NH_3_-Ge N down	−0.62	2.32	0.23	0.10
NH_3_-Si N down	−0.62	2.324 (N-Ge)	0.23	0.10
H_2_-center	−0.15	3.66 (H-Ge)	−0.01	0.02
H_2_O-Ge O down	−0.35	3.00	0.03	0.03
O_2_-center O down	−0.93	2.93 (O-Ge)	−0.24	0

## References

[1] Cui S., Pu H., Wells S.A., Wen Z., Mao S., Chang J., Hersam M.C., Chen J. (2015). Ultrahigh sensitivity and layer-dependent sensing performance of phosphorene-based gas sensors. Nat. Commun..

[2] Tian F., Ru Q., Qiao C., Sun X., Jia C., Wang Y., Zhang Y. (2019). Adsorption desulfurization of model gasoline by metal–organic framework Ni_3_(BTC)_2_. J. Energy Chem..

[3] Smulko J. (2019). Methods of trend removal in electrochemical noise data–Overview. Measurement.

[4] Zhao P., Tang Y., Mao J., Chen Y., Song H., Wang J., Song Y., Liang Y., Zhang X. (2016). One-Dimensional MoS_2_-Decorated TiO_2_ nanotube gas sensors for efficient alcohol sensing. J. Alloy. Compd..

[5] Hashimoto A., Suenaga K., Gloter A., Urita K., Iijima S. (2004). Direct evidence for atomic defects in graphene layers. Nature.

[6] Donarelli M., Ottaviano L. (2018). 2D materials for gas sensing applications: A review on graphene oxide, MoS_2_, WS_2_ and phosphorene. Sensors.

[7] Leenaerts O., Partoens B., Peeters F. (2008). Adsorption of H_2_O, NH_3_, CO, NO_2_, and NO on graphene: A first-principles study. Phys. Rev. B.

[8] Shi Z., Zhang Z., Kutana A., Yakobson B.I. (2015). Predicting two-dimensional silicon carbide monolayers. Acs Nano.

[9] Zhou H., Zhao M., Zhang X., Dong W., Wang X., Bu H., Wang A. (2013). First-principles prediction of a new Dirac-fermion material: Silicon germanide monolayer. J. Phys. Condens. Matter.

[10] Sannyal A., Ahn Y., Jang J. (2019). First-principles study on the two-dimensional siligene (2D SiGe) as an anode material of an alkali metal ion battery. Comput. Mater. Sci..

[11] Juarez-Mosqueda R., Ma Y., Heine T. (2016). Prediction of topological phase transition in X2–SiGe monolayers. Phys. Chem. Chem. Phys..

[12] Vogt P., De Padova P., Quaresima C., Avila J., Frantzeskakis E., Asensio M.C., Resta A., Ealet B., Le Lay G. (2012). Silicene: Compelling experimental evidence for graphenelike two-dimensional silicon. Phys. Rev. Lett..

[13] Ni Z., Liu Q., Tang K., Zheng J., Zhou J., Qin R., Gao Z., Yu D., Lu J. (2011). Tunable bandgap in silicene and germanene. Nano Lett..

[14] Ouyang T., Qian Z., Hao X., Ahuja R., Liu X. (2018). Effect of defects on adsorption characteristics of AlN monolayer towards SO_2_ and NO_2_: Ab initio exposure. Appl. Surf. Sci..

[15] Yue Q., Shao Z., Chang S., Li J. (2013). Adsorption of gas molecules on monolayer MoS_2_ and effect of applied electric field. Nanoscale Res. Lett..

[16] Perdew J.P., Burke K., Ernzerhof M. (1996). Generalized gradient approximation made simple. Phys. Rev. Lett..

[17] Sun X., Yang Q., Meng R., Tan C., Liang Q., Jiang J., Ye H., Chen X. (2017). Adsorption of gas molecules on graphene-like InN monolayer: A first-principle study. Appl. Surf. Sci..

[18] Meng R.-S., Cai M., Jiang J.-K., Liang Q.-H., Sun X., Yang Q., Tan C.-J., Chen X.-P. (2016). First principles investigation of small molecules adsorption on antimonene. IEEE Electron Device Lett..

[19] Wang X.H., Wang D.W., Yang A.J., Koratkar N., Chu J.F., Lv P.L., Rong M.Z. (2018). Effects of adatom and gas molecule adsorption on the physical properties of tellurene: A first principles investigation. Phys. Chem. Chem. Phys..

[20] Ray S. (2016). First-principles study of MoS_2_, phosphorene and graphene based single electron transistor for gas sensing applications. Sens. Actuators B: Chem..

[21] Xia W., Hu W., Li Z., Yang J. (2014). A first-principles study of gas adsorption on germanene. Phys. Chem. Chem. Phys..

[22] Nagarajan V., Chandiramouli R. (2018). Alcohol molecules adsorption on graphane nanosheets–a first-principles investigation. Appl. Surf. Sci..

[23] Ma D., Ju W., Li T., Zhang X., He C., Ma B., Lu Z., Yang Z. (2016). The adsorption of CO and NO on the MoS_2_ monolayer doped with Au, Pt, Pd, or Ni: A first-principles study. Appl. Surf. Sci..

[24] Fu Z., Yang B., Zhang N., Ma D., Yang Z. (2018). First-principles study of adsorption-induced magnetic properties of InSe monolayers. Appl. Surf. Sci..

[25] Qin H., Feng C., Luan X., Yang D. (2018). First-principles investigation of adsorption behaviors of small molecules on penta-graphene. Nanoscale Res. Lett..

[26] Pan W., Qi N., Zhao B., Chang S., Ye S., Chen Z. (2019). Gas sensing properties of buckled bismuthene predicted by first-principles calculations. Phys. Chem. Chem. Phys..

[27] Xie J., Li X., Mao S., Li L., Ke B., Zhang Q. (2018). Effects of structure of fatty acid collectors on the adsorption of fluorapatite (0 0 1) surface: A first-principles calculations. Appl. Surf. Sci..

[28] Yan D., Liu Q., Zeng C., Dong N., Huang Y., Xiao W. (2019). Adsorption of lithium polysulfides on an anatase (1 0 1) and an α-Al_2_O_3_ (0 0 0 1) surface under external electric field with first principles calculations. Appl. Surf. Sci..

[29] Li T., He C., Zhang W. (2018). Electric field improved the sensitivity of CO on substitutionally doped antimonene. Appl. Surf. Sci..

[30] Safari F., Moradinasab M., Fathipour M., Kosina H. (2019). Adsorption of the NH_3_, NO, NO_2_, CO_2_, and CO gas molecules on blue phosphorene: A first-principles study. Appl. Surf. Sci..

[31] Vedaei S.S., Nadimi E. (2019). Gas sensing properties of CNT-BNNT-CNT nanostructures: A first principles study. Appl. Surf. Sci..

[32] Cheng Y., Meng R., Tan C., Chen X., Xiao J. (2018). Selective gas adsorption and I–V response of monolayer boron phosphide introduced by dopants: A first-principle study. Appl. Surf. Sci..

[33] Rumyantsev S., Liu G., Potyrailo R.A., Balandin A.A., Shur M.S. (2013). Selective sensing of individual gases using graphene devices. IEEE Sens. J..

